# Identification of novel mitochondrial mutations in Leber’s hereditary optic neuropathy

**Published:** 2010-04-30

**Authors:** Manoj Kumar, Mukesh Tanwar, Rohit Saxena, Pradeep Sharma, Rima Dada

**Affiliations:** 1 Laboratory for Molecular Reproduction and Genetics, Department of Anatomy, All India Institute of Medical Sciences, Ansari Nagar, New Delhi, India; 2 Dr. R.P. Centre for Ophthalmic Sciences, All India Institute of Medical Sciences, Ansari Nagar, New Delhi, India

## Abstract

**Purpose:**

To screen mitochondrial DNA (mtDNA) variations in Leber hereditary optic neuropathy (LHON).

**Methods:**

Ten LHON patients were selected from neuro-ophthalmology clinics of All India Institute of Medical Sciences (AIIMS), New Delhi, India. Clinical evaluation included slit-lamp biomicroscopy, fundus examination, and neuroimaging. DNA was isolated from whole blood samples. The entire coding region of the mitochondrial genome was amplified by PCR in ten patients and 20 controls. The full mtDNA genome except D-loop was sequenced. All sequences were analyzed against mitochondrial reference sequence NC_012920.

**Results:**

MtDNA sequencing revealed a total of 30 nucleotide variations in the ten LHON patients and 29 in the 20 controls. Of 30 changes, 30.00% (9/30) were nonsynonymous, and the remaining 70.00% (21/30) were synonymous. In controls, a total of five changes were nonsynonymous. Out of the total 14 nonsynonymous changes observed in cases and controls, four (p.A52T in nicotinamide adenine dinucleotide [NADH] dehydrogenase [ND1] protein; p.L128Q in ND2; p.W48R in ATPase6; p.R340H in ND4 protein) were pathogenic. Four patients were positive for either of pathogenic changes. In total, 16.66% (5/30) variations were novel out of which 40.00% (2/5) were nonsynonymous. All novel variations were submitted to the GenBank database, and accession numbers were obtained. Primary LHON mutations in complex I genes have been considered a hallmark feature of LHON patients, and primary LHON mutations were present in two cases in this study. Mutations in complex I genes (ND genes) account for 50%–90% of LHON pedigrees in different ethnic pedigrees. In this study the highest numbers of changes were also present in complex I genes (46.66%; 14/30) followed by complex IV (30.00%; 9/30), complex III (16.66%; 5/30), and then complex V (6.66%; 2/30). Complex I had 5/30 (16.66%) nonsynonymous changes, complex III had 1/30 (3.33%), complex IV had 1/30 (3.33%), and complex V had 2/30 (6.66%) nonsynonymous changes. Nonsynonymous mutations in cytochrome c oxidase (COX) genes have been reported previously in LHON patients. Nonsynonymous mtDNA variations may adversely affect the respiratory chain and impair the oxidative phosphorylation (OXPHOS) pathway, resulting in low ATP production and elevated reactive oxygen species (ROS) levels, which cause oxidative stress. It has previously been reported that oxidative stress (OS) leads to oxidative damage of cellular macromolecules, such as mitochondrial and nuclear DNA, proteins, and lipids along with energy depletion and a local imbalance of calcium homeostasis, resulting in neuronal degeneration. OS is the underlying etiology in several ocular diseases and also plays an essential role in LHON.

**Conclusions:**

A total of five novel mtDNA variations were identified in this study. Nonsynonymous mtDNA variations may adversely affect the respiratory chain and impair the OXPHOS pathway, resulting in low ATP production and elevated ROS levels. OS further damages both nuclear and mtDNA. This preliminary study describes mtDNA sequence variations in a relatively small number of LHON patients of north Indian ethnic origin. However, these results should be confirmed in other populations. Early diagnosis of mtDNA variations and prompt anti-oxidant administration in these cases may delay OS-induced injury to retinal ganglion cells (RGCs) and hence improve visual prognosis.

## Introduction

Leber hereditary optic neuropathy (LHON; OMIM 535000) was first described as a distinctive clinical entity in 1871 by the German ophthalmologist Theodore Leber (1840–1917) [1]. LHON is a maternally inherited disease leading to acute bilateral blindness due to loss of the optic nerve and papillomacular bundle nerve fibers, predominantly in young men [2]. The prevalence is estimated to be 1:50,000. LHON begins generally in young adults, with a mean age of onset between 18 and 35 years. Nearly all patients worldwide carry one of three mitochondrial (mt) DNA pathogenic point mutations at positions 11778/nicotinamide adenine dinucleotide (NADH) dehydrogenase (*ND)4*, 3460/ND1, and 14484/*ND6*, which affect complex I subunit genes [3]. Other pathogenic point mutations have also been described in a minority of patients [4,5]. Although visual failure is the defining feature in this mitochondrial disorder, cardiac arrhythmias, and neurologic abnormalities, such as postural tremor, peripheral neuropathy, nonspecific myopathy [6], and movement disorders [7], have also been reported to be more common in LHON patients compared to controls. Pathogenic mutations in different mitochondrial genes in LHON patients have been reported in previous studies [8-13]. In this preliminary study we investigated a series of patients presenting at ophthalmology clinics with subacute visual failure and who were suspected of having LHON. This study was planned with the aim to screen LHON cases for mtDNA sequence variations by PCR–DNA sequencing.

## Methods

### Clinical examination and selection of cases

After receiving ethical approval from the institutional review board (IRB#00006862; All India Institute of Medical Sciences, Delhi, India), ten clinically diagnosed LHON cases from northern India, presenting at the Dr. R. P. Centre for Ophthalmic Sciences (AIIMS, New Delhi, India), were enrolled for this study only after informed consent. LHON was diagnosed on the basis of a high index of suspicion. Since it is mainly a diagnosis of exclusion, all other causes were considered and ruled out. Detailed family history was taken, including associated peri-ocular pain to differentiate from papillitis, and the history of chronic tobacco or alcohol or chronic systemic medication was noted to rule out toxic optic neuropathy. Family history of any similar episode was noted, and the family tree was charted. Detailed systemic and neurologic examinations were done to check the involvement of other cranial and peripheral nerves. Visual field evaluation, electrophysiological electro-retinogram, and visual-evoked potential were done in all patients. All patients underwent a complete ophthalmic examination, including visual acuity measurement, slit-lamp observation of the anterior segment, indirect ophthalmoscopy, and applanation tonometry. Visual field testing was performed in all patients with good fixation. All patients underwent magnetic resonance imaging (MRI) of the brain and orbit and flourescein angiography.

Hospital records were reviewed, and full neuro-ophthalmologic examinations were performed on all patients. The mean age of onset of symptoms was 24.9 years (all patients were male). No patient reported any drastic changes in their diet or intake of any drug or exposure to any toxic agent/pollutant around the time of visual loss. All patients had normal erythrocyte sedimentation rate, antinuclear antibodies, and syphilis serology. None of the patients reported myotonia, exercise intolerance, palpitations, cardiac conduction abnormalities, oral or genital ulcers, erythema nodosum, or somatic anomalies. Patients were followed in a neuro-ophthalmology clinic. The clinical manifestation of all LHON patients has been tabulated (Table 1).

**Table 1:** Clinical phenotypes of Leber hereditary optic neuropathy patients

**Patient ID**	**Age of onset (in years)**	**Sex**	**Neuro-imaging**	**VA**		**Fundus findings**	**Fields**	
				**OD**	**OS**		**OD**	**OS**
LHON 1	22	M	Normal	CF3ft	CF5ft	diffuse disc pallor	Not possible	Not possible
LHON 2	25	M	Normal	20/80	20/50	diffuse disc pallor	central scotoma	central scotoma
LHON 3	27	M	Normal	CF5ft	20/60	diffuse disc pallor	Not possible	central scotoma
LHON 4	24	M	Normal	HMCF	LP only	diffuse disc pallor	Not possible	Not possible
LHON 5	29	M	Normal	20/100	20/50	Severely diffuse disc pallor	central scotoma	central scotoma
LHON 6	26	M	Normal	20/200	20/100	diffuse disc pallor	central scotoma	central scotoma
LHON 7	28	M	Normal	CF5ft	20/60	temporal disc pallor	Not possible	central scotoma
LHON 8	21	M	Normal	20/60	20/60	Severe diffuse disc pallor	central scotoma	central cecal scotoma
LHON 9	23	M	Normal	20/200	20/100	diffuse disc pallor	central scotoma	central scotoma
LHON 10	24	M	Normal	CF1ft	20/100	diffuse disc pallor	Not possible	central scotoma

Abbrevations: OD represents right eye; OS represents left eye; CF represents counting finger; ft represents distance in feet; HMCF represents hand motions close to face; LP represents light perception; VA represents visual acuity.

A total of 20 ethnically and age-matched normal individuals without any history of ocular disorders was enrolled as controls. All control subjects were AIIMS blood donors who reported no symptomatic metabolic, genetic, or ocular disorders on an extensive questionnaire regarding family history, past medical problems, and current health. The control group for mtDNA sequencing consisted of 20 male individuals (mean age, 23.85±3.34 years). Family information was obtained by history.

### Sample collection and DNA isolation

Peripheral blood samples (5 ml, blood drawn by venipuncture) were collected in EDTA vacutainers (Greiner Bio-one Catalogue no. 455036; Frickenhausen, Germany) after obtaining written consent and stored at −80 °C until further use. DNA was extracted from whole blood samples of all LHON patients and controls, using the phenol chloroform method [14].

### PCR amplification and sequence analysis of the mitochondrial DNA coding region

The entire coding region of the mtDNA was amplified in LHON patients and controls using 24 pairs of primers (Table 2) [15]. PCR amplifications for all primer sets were performed in a 40-μl volume containing 1.0 μl of 20 μM stock solution for each primer (Eurofins Genomics India pvt Ltd, Bangalore, India), 100 ng of genomic DNA, 1 unit of Taq polymerase (Banglore Genei, Bengaluru, Karnataka, India), 0.1 mM of each deoxynucleotide triphosphate (dNTP), and 4 μl of 10X PCR buffer (with 15 mM MgCl_2_) by means of 30 cycles of amplification, each consisting of 30 s denaturation at 94 °C, 30 s annealing at 56 °C, and 1 min extension at 72 °C. Finally, an extension for 5 min at 72 °C was performed.

**Table 2:** Primers used for polymerase chain reaction used for amplification of mitochondrial genome.

**Name**	**Primer sequence**	**Name**	**Primer sequence**
1F.611	CTCCTCAAAGCAATACACTG	13F.8621	TTTCCCCCTCTATTGATCCC
1R.1411	TGCTAAATCCACCTTCGACC	13R.9397	GTGGCCTTGGTATGTGCTTT
2F.1245	CGATCAACCTCACCACCTCT	14F.9230	CCCACCAATCACATGCCTAT
2R.2007	TGGACAACCAGCTATCACCA	14R.10130	TGTAGCCGTTGAGTTGTGGT
3F.1854	GGACTAACCCCTATACCTTCTGC	15F.9989	TCTCCATCTATTGATGAGGGTCT
3R.2669	GGCAGGTCAATTTCACTGGT	15R.10837	AATTAGGCTGTGGGTGGTTG
4F.2499	AAATCTTACCCCGCCTGTTT	16F.10672	GCCATACTAGTCTTTGCCGC
4R.3346	AGGAATGCCATTGCGATTAG	16R.11472	TTGAGAATGAGTGTGAGGCG
5F.3169	TACTTCACAAAGCGCCTTCC	17F.11314	TCACTCTCACTGCCCAAGAA
5R.3961	ATGAAGAATAGGGCGAAGGG	17R.12076	GGAGAATGGGGGATAGGTGT
6F.3796	TGGCTCCTTTAACCTCTCCA	18F.11948	TATCACTCTCCTACTTACAG
6R.4654	AAGGATTATGGATGCGGTTG	18R.12772	AGAAGGTTATAATTCCTACG
7F.4485	ACTAATTAATCCCCTGGCCC	19F.12571	AAACAACCCAGCTCTCCCTAA
7R.5420	CCTGGGGTGGGTTTTGTATG	19R.13507	TCGATGATGTGGTCTTTGGA
8F.5255	CTAACCGGCTTTTTGCCC	20F.13338	ACATCTGTACCCACGCCTTC
8R.6031	ACCTAGAAGGTTGCCTGGCT	20R.14268	AGAGGGGTCAGGGTTGATTC
9F.5855	GAGGCCTAACCCCTGTCTTT	21F.14000	GCATAATTAAACTTTACTTC
9R.6642	ATTCCGAAGCCTGGTAGGAT	21R.14998	AGAATATTGAGGCGCCATTG
10F.6469	CTCTTCGTCTGATCCGTCCT	22F.14856	TGAAACTTCGGCTCACTCCT
10R.7315	AGCGAAGGCTTCTCAAATCA	22R.15978	AGCTTTGGGTGCTAATGGTG
11F.7148	ACGCCAAAATCCATTTCACT	23F.15811	TCATTGGACAAGTAGCATCC
11R.8095	CGGGAATTGCATCTGTTTTT	23R.765	GAGTGGTTAATAGGGTGATAG
12F.7937	ACGAGTACACCGACTACGGC	24F.16420	CACCATTCTCCGTGAAATCA
12R.8797	TGGGTGGTTGGTGTAAATGA	24R.775	AGGCTAAGCGTTTTGAGCTG

Amplified PCR products were purified using a gel/PCR DNA fragments extraction kit (Geneaid Biotech Ltd., Sijhih City, Taiwan). Purified PCR products were sent for sequencing to Molecular Cloning Laboratories (South San Francisco, CA). The full mtDNA genome was sequenced except D-loop, as this part of the mitochondrial genome is a hyper-variable region. All fragments were sequenced in both forward and reverse directions for confirmation of any nucleotide variation. All sequence variants from both LHON patients and controls were compared to the Human Mitochondrial Reference Sequence NC_012920 provided by the National Center for Biotechnology Information (NCBI), using ClustalW2 (multiple sequence alignment program for DNA; European Bioinformatics Institute, Wellcome Trust Genome Campus, Hinxton, Cambridge, UK).

### Computational assessment of missense mutations

Two homology-based programs, Polymorphism Phenotyping (PolyPhen) and Sorting Intolerant From Tolerant (SIFT) analysis tool, were used to predict the functional impact of missense changes identified in this study. PolyPhen structurally analyzes an amino acid polymorphism and predicts whether that amino acid change is likely to be deleterious to protein function [16,17]. PolyPhen scores of >2.0 indicate the polymorphism is probably damaging to protein function. Scores of 1.5–2.0 are possibly damaging, and scores of <1.5 are likely benign. SIFT is a sequence homology-based tool that sorts intolerant from tolerant amino acid substitutions and predicts whether an amino acid substitution in a protein will have a phenotypic effect [18]. SIFT is based on the premise that protein evolution is correlated with protein function. Positions with normalized probabilities less than 0.05 are predicted to be deleterious and those greater than or equal to 0.05 are predicted to be tolerated in the case of SIFT.

The substitution may occur at a specific site, e.g., active or binding, or in a nonglobular, e.g., transmembrane, region. PolyPhen uses the predicted hydrophobic and transmembrane (PHAT) matrix score to evaluate the possible functional effect of a substitution in the transmembrane region. At this step Polyphen memorizes all positions that are annotated in the query protein as BINDING, ACT_SITE, LIPID, or METAL. At a later stage, if the search for a homologous protein with known three-dimensional structure is successful, PolyPhen is checked whether the substitution site is in spatial contact with these critical residues. Positions important for function should be conserved in an alignment of the protein family, whereas unimportant positions should appear diverse in an alignment.

## Results

MtDNA sequencing revealed a total of 30 nucleotide variations in the ten LHON patients, of which 30.00% (9/30) were nonsynonymous (Table 3), and 29 nucleotide changes in 20 controls, of which 17.24% (5/29) were nonsynonymous (Table 4). Of the 30 changes 30.00% (9/30) were nonsynonymous and the remaining 70% (21/30) were synonymous. Of the total changes 96.66% were transitions and the remaining were transversions. In total, 16.66% (5/30) variations were novel, out of which 40.00% (2/5) were nonsynonymous. Twenty-five other mtDNA sequence variations (see the MITOMAP and DB) were also identiﬁed in patients. The highest number of changes were present in complex I genes (46.66%; 14/30), followed by complex IV (30%; 9/30), then complex III (16.66%; 5/30), then complex V (6.66%; 2/30) genes. Complex I had 5/30 (16.66%) nonsynonymous changes, complex III had 1/30 (3.33%), complex IV had 1/30 (3.33%), and complex V had 2/30 (6.66%) nonsynonymous changes. All nucleotide variations identified in the current study were homoplasmic. All novel variations were submitted to the GenBank database, and accession numbers were obtained (Table 3).

**Table 3:** Mitochondrial DNA sequence changes in Leber hereditary optic neuropathy patients.

**Sample number**	**Nucleotide substitution**	**Codon Change**	**Amino acid change**	**Locus**	**Base substitution type**	**Type of mutation**	**Polyphen/SIFT score**	**Pathogenic**	**GenBank accession number if novel**
1	*3460 G>A	GCC>ACC	p.A52T	ND1	Transition	NS	1.646/0.00	YES	NA
2	4852 T>A	CTG>CAG	p.L128Q	ND2	Transversion	NS	1.951/0.00	YES	GU197845
3	4944 A>G	ATC>GTC	p.I159V	ND2	Transition	NS	0.468/0.29	NO	NA
4	5004 T>C	TTA>CTA	p.L179L	ND2	Transition	SYN	NA	NA	NA
5	6032 G>A	CAG>CAA	p.Q43Q	CO1	Transition	SYN	NA	NA	NA
6	6320 T>C	CCT>CCC	p.P139P	CO1	Transition	SYN	NA	NA	NA
7	6734 G>A	ATG>ATA	p.M277M	CO1	Transition	SYN	NA	NA	NA
8	6908 T>C	TCT>TCC	p.S335S	CO1	Transition	SYN	NA	NA	NA
9	7702 G>A	CTG>CTA	p.L39L	CO2	Transition	SYN	NA	NA	NA
10	8155 G>A	GGG>GGA	p.G190G	CO2	Transition	SYN	NA	NA	NA
11	8668 T>C	TGA>CGA	p.W48R	ATP6	Transition	NS	2.734/0.03	YES	NA
12	8684 C>T	ACC>ATC	p.T53I	ATP6	Transition	NS	0.219/1.00	NO	NA
13	9254 A>G	TGA>TGG	p.W16W	CO3	Transition	SYN	NA	NA	NA
14	9767 C>T	ACC>ACT	p.T187T	CO3	Transition	SYN	NA	NA	NA
15	9966 G>A	GTC>ATC	p.V254I	CO3	Transition	NS	0.293/0.46	NO	NA
16	10238 T>C	ATT>ATC	p.I60I	ND3	Transition	SYN	NA	NA	NA
17	10256 T>C	GAT>GAC	p.D66D	ND3	Transition	SYN	NA	NA	NA
18	10400 C>T	ACC>ACT	p.T114T	ND3	Transition	SYN	NA	NA	NA
19	10589 G>A	CTG>CTA	p.L40L	ND4L	Transition	SYN	NA	NA	NA
20	*11778 G>A	CGC>CAC	p.R340H	ND4	Transition	NS	2.608/0.00	YES	NA
21	12348 C>T	CAC>CAT	p.H4H	ND5	Transition	SYN	NA	NA	GU197843
22	12477 T>C	AGT>AGC	p.S47S	ND5	Transition	SYN	NA	NA	NA
23	12681 T>C	AAT>AAC	p.N115N	ND5	Transition	SYN	NA	NA	NA
24	12732 T>C	GTT>GTC	p.V132V	ND5	Transition	SYN	NA	NA	NA
25	13151 T>C	CTA>CCA	p.L272P	ND5	Transition	NS	0.175/0.21	No	GU197844
26	14783 T>C	TTA>CTA	p.L13L	CYB	Transition	SYN	NA	NA	NA
27	14950 C>T	CAC>CAT	p.H68H	CYB	Transition	SYN	NA	NA	GU197846
28	15067 T>C	TTT>TTC	p.F107F	CYB	Transition	SYN	NA	NA	NA
29	15110 G>A	GCA>ACA	p.A122T	CYB	Transition	NS	0.401/0.65	NO	NA
30	15493 C>T	CTC>CTT	p.L249L	CYB	Transition	SYN	NA	NA	GU197847

Abbrevations: *Primary Leber hereditary optic neuropathy (LHON) mutations, SYN represents synonymous, NS represents Not synonymous, NA represents Not applicable Transition represents It is a mutation in which a purine/pyrimidine base pair is replaced with a base pair in the same purine/pyrimidine relationship (A:T>G:C or C:G>T:A). Transversion represents It is a mutation in which a purine/pyrimidine replaces a pyrimidine/purine base pair or vice versa (G:C>T:A or C:G, or A:T>T:A or C:G).

**Table 4:** Mitochondrial DNA sequence changes in controls

**Sample number**	**Nucleotide substitution**	**Codon Change**	**Amino acid change**	**Locus**	**Change in protein**	**Type of Mutation**	**PolyPhen/ SIFT score**	**Pathogenecity**
1	3591G>A	CTG>CTA	Thr>Thr	ND1	p.T95T	SYN	NA	NA
2	3915G>A	GGG>GGA	Gly>Gly	ND1	p.G203G	SYN	NA	NA
3	3918G>A	GAG>GAA	Glu>Glu	ND1	p.E204E	SYN	NA	NA
4	3933A>G	TCA>TCG	Ser>Ser	ND1	p.S209S	SYN	NA	NA
5	4093A>G	ACC>GCC	Thr>Ala	ND1	p.T263A	NS	0.476/0.38	No
6	4793A>G	ATA>ATG	Met>Met	ND2	p.M108M	SYN	NA	NA
7	5351A>G	CTA>CTG	Leu>Leu	ND2	p.L294L	SYN	NA	NA
8	6305G>A	GGG>GGA	Gly>Gly	CO1	p.G134G	SYN	NA	NA
9	6962G>A	CTG>CTA	Thr>Thr	CO1	p.T353T	SYN	NA	NA
10	7738T>C	ACT>ACC	Thr>Thr	CO2	p.T51T	SYN	NA	NA
11	7762G>A	CAG>CAA	Gln>Gln	CO2	p.Q59Q	SYN	NA	NA
12	8143T>C	GCT>GCC	Ala>Ala	CO2	p.A186A	SYN	NA	NA
13	8251G>A	GGG>GGA	Gly>Gly	CO2	p.G222G	SYN	NA	NA
14	8503T>G	AAT>AAG	Asp>Lys	ATP8	p.N46K	NS	0.090/1.00	No
15	8584G>A	GCA>ACA	Ala>Thr	ATP6	p.A20T	NS	0.362/0.19	No
16	8650C>T	CTA>TTA	Leu>Leu	ATP6	p.L42L	SYN	NA	NA
17	8718A>G	AAA>AAG	Lys>Lys	ATP6	p.K64K	SYN	NA	NA
18	8886G>A	AAG>AAA	Lys>Lys	ATP6	p.K120K	SYN	NA	NA
19	10310G>A	CTG>CTA	Thr>Thr	ND3	p.T84T	SYN	NA	NA
20	11467A>G	TTA>TTG	Leu>Leu	ND4	p.L236L	SYN	NA	NA
21	11914G>A	ACG>ACA	Thr>Thr	ND4	p.T385T	SYN	NA	NA
22	12372G>A	CTG>CTA	Tyr>Tyr	ND5	p.T12T	SYN	NA	NA
23	12406G>A	GTT>ATT	Val>Ile	ND5	p.V24I	NS	0.299/0.72	No
24	12486C>T	CCC>CCT	Pro>Pro	ND5	p.P50P	SYN	NA	NA
25	12498C>T	TTC>TTT	Phe>Phe	ND5	p.F54F	SYN	NA	NA
26	12561G>A	CAG>CAA	Gln>Gln	ND5	p.Q75Q	SYN	NA	NA
27	13204G>A	GTC>ATC	Val>Ile	ND5	p.V290I	NS	0.710/1.00	No
28	15172G>A	GGG>GGA	Gly>Gly	CYB	p.G142G	SYN	NA	NA
29	15217G>A	GGG>GGA	Gly>Gly	CYB	p.G157G	SYN	NA	NA

Abbreviations; SYN represents synonymous; NS represents non-synonymous; ND1 represents NADH dehydrogenase subunit 1; ND2 represents NADH dehydrogenase subunit 2; ND3 represents NADH dehydrogenase subunit 3; ND4 represents NADH dehydrogenase subunit 4; ND5 represents NADH dehydrogenase subunit 5; CO1 represents cytochrome c oxidase I ; CO2 represents cytochrome c oxidase II; ATPase6- represents ATP synthase subunit a (F-ATPase protein 6) ; ATPase8 represents ATP synthase protein 8 ; CYB represents cytochrome B; NA represents Not applicable.

### Sorting Intolerant From Tolerant and Polymorphism Phenotyping analysis

SIFT and PolyPhen analysis of all 14 nonsynonymous changes (9+5) from cases and controls revealed four pathogenic changes (p.A52T in ND1 protein; p.L128Q in ND2; p.W48R in ATPase6; p.R340H in ND4 protein; Table 3). Four patients (LHON 5–8, Table 5) were positive for at least one of these pathologic mtDNA nucleotide changes, but none of the controls harbored any pathogenic nucleotide change (Table 4). Patient and gene-wise distribution of mtDNA sequence changes have been tabulated (Table 5).

**Table 5:** Patient and gene wise distribution of mitochondrial DNA variations

**Patient ID**	***ND1***	***ND2***	***CO1***	***CO2***	***ATPase6***	***CO3***	***ND3***	***ND4L***	***ND4***	***ND5***	***CYB***
LHON 1	—	p.I159V, p.L179L	p.S335S	—	—	p.W16W	—	—	—	—	p.F107F
LHON 2	—	p.I159V	—	—	—	—	p.T114T	p.L40L	—	p.S47S, p.N115N	—
LHON 3	—	—	—	—	p.T53I	—	—	—	—	—	p.A122T, p.L249L
LHON 4	—	—	p.P139P	—	—	—	—	—	—	p.V132V, p.H4H	—
LHON 5	—	—	—	—	p.W48R	—	p.T114T	—	p.R340H	—	p.H68H
LHON 6	—	p.L128Q	—	—	—	—	—	—	—	—	p.L13L
LHON 7	—	p.L128Q	p.Q43Q	—	p.T53I	p.T187T	—		—	—	—
LHON 8	p.A52T	—	p.M277M	p.L39L, p.G190G	—	p.W16W, p.V254I	p.I60I, p.D66D	—	—	p.L272P	—
LHON 9	—	—	p.P139P	—	p.T53I	p.V254I	—	—	—	p.L272P	—
LHON 10	—	p.I159V	—	—	—	—	p.I60I	—	—	—	p.A122T

Abbreviations: LHON represents Leber hereditary optic neuropathy, ND1 represents NADH dehydrogenase subunit 1, ND2 represents NADH dehydrogenase subunit 2, ND3 represents NADH dehydrogenase subunit 3, ND4 represents NADH dehydrogenase subunit 4, ND5 represents NADH dehydrogenase subunit 5, CO_2_ represents cytochrome c oxidase II, CYB represents cytochrome B.

## Discussion

In this preliminary pilot study, we enrolled ten LHON patients who experienced acute or subacute, bilateral, persistent, optic neuropathies characterized by central visual loss that occurred simultaneously or sequentially within a period of 1 year. Leber described a maternal inheritance pattern; however, some patients with primary LHON mutations deny having a family history.

LHON-associated mtDNA mutations have been identified in various ethnic populations [19,20]. Mutations in complex I genes (ND genes) account for 50%–90% of LHON pedigrees in different ethnic pedigrees [11,13,21,22]. Primary LHON mutations have been considered a hallmark feature of LHON patients, and patients without primary LHON mutations have also been reported [23,24]. In this study 46.66% variations were reported in complex I (ND group of genes) genes, of which three were novel changes. Three pathogenic mutations (p.A52T in *ND1*; p.L128Q in *ND2*; and p.R340H in *ND4*) were present in complex I genes. Primary LHON mutation 3460G>A (p.A52T) was present in one patient (LHON 8), 11778G>A (p.R340H) was present in one patient (LHON 5), and 14484T>C (p.M64V in *ND6*) was absent in our patients.

Altered ubiquinone binding to complex I has been previously shown in the presence of several LHON-causing mutations. Decreased affinities for ubiquinone or the complex I inhibitor rotenone have been shown in the 3460G>A (p.A52T) and 11778G>A (p.R340H) mutations [25-28]. Substrate inhibition at higher concentrations of ubiquinone has previously been observed in the mitochondrial membrane preparations from Ebstein-Barr virus-transformed cell lines carrying the 3460G>A (p.A52T) or 14459G>A (p.A72V in *ND6*) mutations [25,29]. No primary LHON mutation or pathogenic mutation was present in control subjects. The age of onset symptoms did not differ significantly in patients with and without pathogenic mutations in the current study.

In the current study two patients (LHON 8 and LHON 5) had primary LHON mutations (3460G>A [p.A52T]; 11778G>A [p.R340H]). But none of the patients with primary LHON mutations reported a multigenerational history compatible with maternal inheritance. Two patients (LHON 6 and LHON 7) had pathogenic mtDNA sequence changes other than primary LHON mutations, while others had no suspicious mtDNA changes. Patients with no obvious mtDNA abnormalities might have no mitochondrial disease or they might have mtDNA abnormalities isolated to the optic nerve, conceptually similar to mitochondrial myopathies [30]. However, these patients may have elevated oxidative stress comparable to patients with primary LHON mutations [31]. Previous studies have suggested similar patients with LHON-like optic neuropathies but no identified pathogenic mtDNA mutations after sequencing all [24] or part [32] of the mitochondrial coding region. The phenotypic expression of the primary LHON mutations is very complex. Only about one-third of individuals harboring one of these three mutations eventually develops LHON, and the penetrance varies among different families [33-35]. Therefore, identification of other factors affecting LHON penetrance would be of value in elucidating the pathophysiology of retinal neuron loss as well as in searching for clues that might relieve visual loss or prevent the onset of LHON. Many factors, such as mtDNA background, heteroplasmy of the mtDNA mutation, nuclear gene(s), and environmental factors, have been shown to play active roles in the phenotypic expression of LHON [36-38].

In this study one nonsynonymous sequence change and four synonymous sequences in the cytochrome c oxidase III (*COIII)* gene were also identified of which two were novel. Nonsynonymous mutations in the *COIII* gene have been reported previously in LHON patients [12,39]. Other human diseases associated with cytochrome c oxidase (*COX)* mutations include primary congenital glaucoma (PCG), primary open angle glaucoma (POAG), and Leigh syndrome [15,40,41].

In this study one patient (LHON 10) had a nonsynonymous sequence change in cytochrome *b* (complex III). Nonsynonymous mutations in the cytochrome *b* gene has previously been described in LHON [42]. Two nonsynonymous changes in *ATPase6* (p.T53I and p.W48R) were also identified, one of which (p.W48R) was pathogenic and present in one patient (LHON 5). *ATPase6* mutations in LHON patients have been described previously [43]. Mutations in *ATPase6* have been reported in PCG, POAG, neuropathy, ataxia, retinitis pigmentosa, and mitochondrial DNA-associated Leigh Syndrome patients [15,40,44,45]. In this study eight patients had no primary LHON mutations. However, two other pathogenic mutations were present in three patients (Table 5).

An intriguing feature of LHON is that only 50% of males and 10% of females who harbor one of the three primary mutations actually develop the optic neuropathy. This incomplete penetrance and predilection for males to lose vision imply that additional genetic and/or environmental factors must modulate the phenotypic expression of LHON. No generally accepted measures have been shown to either prevent or delay the onset of blindness in LHON. The long-term management of visually impaired patients remains supportive, with provision of visual aids and registration with the relevant social services.

Many mechanisms have been studied and proposed as the bases for the pathogenesis of mitochondrial optic neuropathies, including bioenergetic failure, oxidative stress, glutamate toxicity, abnormal mitochondrial dynamics and axonal transport, and susceptibility to apoptosis [3]. It has been well established that optic atrophy is a very common and sometimes the singular pathological feature in mitochondrial disorders [41]. Several human diseases have been associated with mtDNA mutations, indicating that dysfunction of the components of oxidative phosphorylation encoded by the mitochondrial genome can be deleterious [46]. Abnormalities in mtDNA have proven to be associated with LHON [40], POAG, pseudoexfoliation glaucoma (PEG), primary angle-closure glaucoma (PACG), PCG, and other spontaneous optic neuropathies [15,47-50]. It is generally agreed that there are two main sites in the respiratory chain where superoxide anions are generated, viz., complex I and complex III [51,52]. In the current study complex I genes had 16.66% nonsynonymous sequence changes, and of these three were pathogenic mutations.

The distribution of high numbers of mitochondria in the optic nerve head reflects the high energy requirement of the human optic nerve head. Neurons, because of their high energy requirement, are heavily dependent on mitochondria for survival [53]. Any malfunction of the mitochondrial electron transport chain results in an excessive generation of free radicals and low ATP production. It has previously been reported that oxidative stress (OS) leads to oxidative damage of cellular macromolecules, such as mitochondrial and nuclear DNA, proteins, and lipids, along with energy depletion and a local dysregulation of calcium homeostasis, resulting in neuronal degeneration [54]. OS is the underlying etiology in several ocular diseases, such as glaucoma, LHON, proliferative vitreoretinopathies, and cataract [40,55-57]. It has been established that pathogenic mitochondrial mutations can cause mitochondrial dysfunction and enhance OS, which in turn lead to apoptosis in affected tissue and primary culture of human cells that harbor mtDNA mutations [58]. Nonsynonymous mtDNA alterations may lead to mitochondrial dysfunction, which leads to reduced mitochondrial respiration, OS, damage to mtDNA, altered mitochondrial morphology, alterations in mitochondrial fission and fusion, and ultimately the cell’s demise. Oxidative stress-induced mtDNA damage has also been reported in other diseases, such as premature ovarian insufficiency [59]. LHON and LHON-like optic neuropathies might approach 20 per 100,000 individuals [60], and this diagnosis may be responsible for as much as 10% of blindness in individuals under age 65 [61].

This study describes mtDNA sequence variations in a relatively small number of LHON patients of north Indian ethnic origin. Nonsynonymous mitochondrial variations adversely affect oxidative phosphorylation, resulting in decreased mitochondrial respiration and increased free radical production [62]. However, these results should be confirmed by larger studies in other populations. Knowledge of mtDNA mutations and/or mitochondrial dysfunction in LHON may lead to a better understanding of optic atrophy in LHON. Novel approaches are now available for studying mitochondrial disease in the eye, and a novel in vitro treatment has already been devised for the metabolic defect of at least one mtDNA mutation in LHON [63]. Further work and ideas must be forthcoming to realistically treat mtDNA disease or prevent the transmission of mtDNA disease to future generations. Early diagnosis and prompt management by anti-oxidants may delay the progression of disease.

### Conclusion

A total of five novel mtDNA variations were identified in this study, of which one was pathogenic. Nonsynonymous mtDNA variations may adversely affect the respiratory chain and impair the OXPHOS pathway, resulting in low ATP production and elevated ROS levels. OS further damages both nuclear and mtDNA. This preliminary study describes mtDNA sequence variations in a relatively small number of LHON patients of north Indian ethnic origin. However, these results should be confirmed in other populations.
